# Supplementary material for: Crowdsourcing airway annotations in chest computed tomography images
Source: PLoS One. 2021 Apr 22;16(4):e0249580. doi: 10.1371/journal.pone.0249580 (PMC8062042; doi:10.1371/journal.pone.0249580)
Supplement: Attachment — Submitted filename: response_letter 12.02.2021.pdf [file pone.0249580.s001.pdf]

Response letter [PONE-D-20-37113]

Veronika Cheplygina[1*], Adria Perez-Rovira[2,3], Wieying Kuo[3,4], Harm A. W. M. Tiddens[3,4], Marleen de Bruijne[2,5]

1 IMAG/e, Department of Biomedical Engineering, Eindhoven University of Technology, Eindhoven, The Netherlands
2 Biomedical Imaging Group Rotterdam, Department of Radiology and Nuclear Medicine, Erasmus MC - University Medical Center Rotterdam, Rotterdam, The Netherlands
3 Department of Pediatric Pulmonology and Allergology, Erasmus MC - Sophia Children's Hospital, Rotterdam, The Netherlands
4 Department of Radiology and Nuclear Medicine, Erasmus MC - University Medical Center Rotterdam, Rotterdam, The Netherlands
5 Machine Learning Section, Department of Computer Science, University of Copenhagen, Denmark

* v.cheplygina@tue.nl

Summary

We prepared a revision of our paper Crowdsourcing Airway Annotations in Chest Computed Tomography Images [PONE-D-20-37113].

For readability, editor/reviewer comments are in **bold**, and text that has been added or changed, when quoted in our response, is in blue. In the revised manuscript additions are in blue as well. For moderate and major changes, the modified text is pasted in the response letter.

Comments Editor

The paper has a considerably interest in the field, easy to follow, with some minor concerns raised by the reviewers.

Thank you for these comments and for taking the time to edit our manuscript.

When submitting your revision, we need you to address these additional requirements.

1) Please ensure that your manuscript meets PLOS ONE's style requirements, including those for file naming.

We reviewed the style guide and made the following changes:

- Renamed the figures to Fig1. etc
- Changed section titles to sentence case
- Included Excel based versions of the tables

2) Please provide additional details regarding participant consent. In the Methods section, please ensure that you have specified how consent was obtained and how the study met relevant personal data and privacy

laws. If data were collected anonymously, please include this information. Participants could in our case be the patients whose chest scans were analyzed, or the workers who annotated the images. For both groups we added more information in the Methods section. For the patients, we added:

These scans have been collected and anonymized for previous studies, and approved to be used for further research. The anonymized scans were shared with us, including the age and sex of the subject, whether the subject had cystic fibrosis, and several measures related to lung capacity and airway size.

For the workers, we added:

Workers consented to doing the task when accepting it on Amazon MT. Due to the non-graphic nature and anonymous character of the airway slices, no other specific approval was obtained. For each result, we recorded an anonymized ID of the worker and the coordinates of the annotations. No other information about the workers was recorded.

3) Please include a copy of Tables 1 amp; 2 which you refer to in your text.

Tables were included in-line in the text of the initial manuscript, in accordance with the guidelines. We slightly improved the formatting of the tables, so they might be more visible. We now also included Excel files for all tables.

Comments Reviewer 1

This paper presents an experiment of crowdsourcing for medical image annotation, specifically annotating airway and airway wall from 2D resampled CT scans. I would say this is a valid study and whether crowdsourcing can be used to for medical applications is an interesting topic. Overall the manuscript is clear and easy to follow. Thank you for the comments and taking the time to review our manuscript.

Some details that need to be considered in revision:

- "is our earlier study [2] where we described our experiences with crowdsourcing airway measurements." [2] does not seem to be the correct citation, guess it should be [9]?

The reference should indeed be to Cheplygina et al paper from 2016 (the numbers have been updated after adding further references). We are unfortunately not able to replicate this error, perhaps an outdated version of the .bbl file was included.

- What is the voxel size of the CT images? What is the physical length of 50x50 voxels?

- The 1026 image patches, how are they distributed along the airway tree? Which generation of branch do they belong to? It will help to understand the approximate size of the airways, by comparing with the dimensions from the last question.

To address both questions above, we added a paragraph to the Methods section:

The voxel size was $0.5508 \times 0.5508 \times 0.6000$ mm. Each scan contained a number of airways of different generations from the 2nd to the 14th. The most common generations were the 6th (23.7%), 7th (20.0%), 5th (17.7%) and 8th (13.9%).

- Inner and outer diameter, since they are ellipses, which "diameter" is used? Major? Minor? Average of major and minor? or average of all?

We have added these details to the Methods section:

Note that both the workers and the experts place ellipses in slices with roughly perpendicular airways, resulting in almost-circular ellipses. For the workers we have

Table 1. Pearson correlations between the expert and the crowd with different combining methods, and between two experts.

Method	vs Expert 1				vs Expert 2			
	inner	outer	wap	wtr	inner	outer	wap	wtr
None	0.803	0.697	0.426	0.424	0.808	0.686	0.469	0.466
Random	0.803	0.701	0.421	0.418	0.813	0.679	0.489	0.481
Median	0.844	0.769	0.572	0.565	0.850	0.746	0.661	0.649
Best	0.858	0.896	0.585	0.590	0.858	0.842	0.583	0.570
Expert 1					0.964	0.925	0.701	0.687

both the major and minor diameters available, but the experts only record the area. For comparisons between the two, we therefore had to assume circular airways.

- Just to confirm, considering the "not seeing an airway" cases, for the input image patches, airway is always visible right? Is there a case that everyone marks as "not seeing"?

This is correct, as we write in the methods one author with experience with CT scans reviewed the patches to only retain ones with a visible airway. We reviewed several cases marked by all or most workers as "cannot see an airway" and added the following findings to our results:

We visually examined several cases where all or most workers indicated not seeing an airway. These appeared to be difficult cases due to low contrast and/or only part of the airway wall being visible (but not so much the size of the airway). While a trained observer would identify these as airways, it is not unexpected that the workers were not able to do so.

- Table 2, Expert 1 and 2 seems to have fairly good agreement, still, it may be helpful to also give the coor number for three combination against Expert 2 (and maybe further against the average number of 1&2)

We added the agreement with expert 2 to Table 2 as well, for convenience a copy of the table is included below. We did not add combinations of experts 1 and 2 as multiple options (minimum, maximum) would be possible, however any of these can be easily generated via notebook linked on our Github page `https://www.github.com/adriapr/crowdairway`.

- Maybe out-of-scope for this paper, but it would be interesting to see how good a SOTA automated algorithm can achieve on the same samples.

Defining a SOTA algorithm is a bit complex here, because ground truth is rare (which was also part of the inspiration for our study). We did not address such a comparison here, however we did add a discussion paragraph on how the annotations may be used for training such algorithms:

Our study was inspired by the lack of annotated datasets for machine learning, however, we focused on evaluating the quality of the annotations alone. There are indications that lower quality labels may still be useful for training machine learning algorithms. For example, combinations of expert and crowdsourced labels have shown to be more effective in cases where the crowdsourced labels alone were not sufficient [1]. More generally, weakly-supervised learning with approximate annotations such as bounding boxes shows that labels do not need to be precise to add value during training [2].

Comments Reviewer 2

The authors facilitated Amazon Mechanical Turk to crowdsource airway annotations from chest CT slices. Annotations from multiple workers were analyzed against the annotations provided by two experts. Almost half of the annotations from workers were identified as invalid due to insufficient instructions and/or issues in experimental design. These issues are discussed in the manuscript and the manuscript provides enough information to design a robust crowdsourcing platform for chest CT airway annotations.

Thank you for the comments and taking the time to review our manuscript.

The authors used crowdsourcing to annotate datasets to machine learning algorithms, as indicated in the abstract. However, experiments analyzing the effect of using annotated dataset generated by workers in ML algorithms were not performed or discussed. It is not clear if nonexpert annotated "good-quality" dataset can be of any use in ML model development or not. Please elucidate.

Datasets for machine learning algorithms were indeed our initial inspiration to look into crowdsourcing. However the annotation process has turned up several issues we did not anticipate, and then into an entire study, which we felt was valuable to report about. For this reason we do not report on performances of algorithms trained on this data. However, we have added our hypotheses (based on prior research) about this possibility in the discussion:

Our study was inspired by the lack of annotated datasets for machine learning, however, we focused on evaluating the quality of the annotations alone. There are indications that lower quality labels may still be useful for training machine learning algorithms. For example, combinations of expert and crowdsourced labels have shown to be more effective in cases where the crowdsourced labels alone were not sufficient [1]. More generally, weakly-supervised learning with approximate annotations such as bounding boxes shows that labels do not need to be precise to add value during training [2].

Page 7 2nd paragraph of the Discussion Section. Please add the % sign after 57.2

Fixed

The manuscript is easy to follow, with issues on the Figure numbers which could be mitigated by using the Figure number as a filename.

Fixed

References

1. Albarqouni S, Baur C, Achilles F, Belagiannis V, Demirci S, Navab N. AggNet: Deep Learning From Crowds for Mitosis Detection in Breast Cancer Histology Images. IEEE Transactions on Medical Imaging. 2016 May;35(5):1313–1321.

2. Cheplygina V, de Bruijne M, Pluim JP. Not-so-supervised: a survey of semi-supervised, multi-instance, and transfer learning in medical image analysis. Medical image analysis. 2019;54:280–296.
